# The Evaluation of Pain with Nociceptive and Neuropathic Characteristics from Three Different Perspectives in Amyotrophic Lateral Sclerosis Patients: A Case Controlled Observational Study in Southwestern China

**DOI:** 10.1155/2021/5537892

**Published:** 2021-07-30

**Authors:** Ran An, Yan Li, Xianghua He, Cheng Li, Xin Li, Yanming Xu, Chengqi He

**Affiliations:** ^1^Department of Rehabilitation Medicine, West China Hospital, Sichuan University, China; ^2^Key Laboratory of Rehabilitation Medicine in Sichuan Province, Chengdu, China; ^3^Department of Central Transportation Center, West China Hospital, Sichuan University, Chengdu, China; ^4^Department of Neurology, West China Hospital, Sichuan University, Chengdu, China

## Abstract

**Background:**

Pain was considered a common and neglected symptom in amyotrophic lateral sclerosis (ALS) and had a substantial impact on the quality of life of ALS patients and their caregivers. However, pain in ALS was mainly evaluated from the perspective of nociceptive pain; only three studies referred to neuropathic pain in ALS, and there has been yet no study considering the neuropathic pain characteristics in ALS patients from China. Therefore, the purpose of our study was to determine characteristics of pain (nociceptive pain and neuropathic pain) by three different types of questionnaires. The correlation between pain and clinical parameters in ALS patients was also evaluated.

**Methods:**

Patients were eligible if they fulfilled the criteria of probable and definitive ALS according to the revised El Escorial criteria. Healthy normal controls, matched to ALS patients by age and gender, were recruited. Pain was evaluated by numerical pain rating scale (NRS), Brief Pain Inventory (BPI), and Douleur Neuropathique-4 (DN4) in ALS patients and controls. Physical status of ALS patients was evaluated with ALS Functional Rating Scale-revised (ALSFRS-R).

**Results:**

65 patients with sporadic ALS and 100 healthy normal controls in Southwestern China were included. Pain in the preceding week was more frequently reported by patients with ALS (30, 46.2%) than controls (36, 36%) (*p* = 0.193). DN4 score⩾4 was found in three ALS patients and one control (*p* = 0.480). Ten ALS patients (33.3%) and twenty-eight controls (77.8%) (*p* < 0.001) received therapy for pain. ALS patients with a DN4 score ≥ 4 had a longer disease duration and a higher PSI and PII score than ALS cases reporting nociceptive pain (*p* = 0.041, 0.048, and 0.027, respectively). Pain mainly interfered with ALS patients' mood, enjoyment of life, and the Pain Interference Index (PII) score.

**Conclusions:**

Our findings indicated that pain in our ALS cohorts was insufficiently treated and interfered with patients' mood and enjoyment of life. Most notably, we found that ALS patients with a DN4 score⩾4 may have a longer disease duration and a higher PSI and PII score than ALS patients reporting nociceptive pain, which has never been reported, strongly deserving further validation.

## 1. Introduction

Amyotrophic lateral sclerosis (ALS) is a neurodegenerative disorder of unknown etiology, characterized by the progressive loss of upper and lower motor neurons, causing weakness and atrophy of upper and lower limbs, dysphagia, and dysarthria, which eventually resulted in death due to respiratory failure typically within 2–4 years from onset [1]. Previously, pain has been considered relatively rare in ALS patients. Since an increasing number of studies about pain have been reported worldwide, pain was considered a common and neglected symptom in ALS, with the frequency of pain varying from less than 15% up to 85% [1–19]. Considering the substantial impact of pain on the quality of life of ALS patients and their caregivers, the identification and evaluation of pain in ALS should not be disregarded. Guidelines for ALS treatment also reported that pain may be present in ALS patients and should be treated [20]. However, due to the scarcity of research data, pain in ALS was still frequently underestimated and insufficiently treated. In addition, to the best of our knowledge, pain in ALS was mainly evaluated from the perspective of nociceptive pain with various methods or questionnaires in different studies, including visual analog scale (VAS) [14], numerical pain rating scale (NRS) [5], Brief Pain Inventory (BPI) [2, 3, 7, 9, 10, 16, 19], Pain Detect Questionnaire [3, 7], Wong-Baker Faces Pain Rating Scale (WBS) [3, 7], and McGill Pain Questionnaire (MPQ) [3, 7]. Besides, there were only three studies about neuropathic pain in ALS from American, France, and Brazil populations by using Douleur Neuropathique-4 (DN4) or Neuropathic Pain Scale (NPQ) [10, 11, 15]. In China, pain characteristics in ALS patients (*n* = 89) was only assessed in a northern city of China, however, without evaluation of the characteristics of neuropathic pain. That is, up to now, there has been no study considering the neuropathic pain characteristics of ALS patients in China.

Therefore, the objective of our study was to determine prevalence, severity, site, type of pain (nociceptive pain and neuropathic pain), and its treatment, interference with activities by three different types of questionnaires (NRS, single-dimensional scale; BPI, multidimensional scale; DN4, neuropathic pain scale) in patients with sporadic ALS and healthy controls from Southwestern China. The correlation between pain and clinical parameters in ALS patients was also evaluated.

## 2. Participants and Methods

### 2.1. Participants

Patients were eligible if they fulfilled the criteria of probable and definitive ALS according to the revised El Escorial criteria [21]. Patients who had signs and symptoms of (frontotemporal) dementia were excluded. All consecutive ALS patients were seen and diagnosed by author Yanming Xu (an expert specialized in neurodegenerative diseases) from the Department of Neurology in our hospital.

Healthy normal controls, matched to ALS patients by age and gender and free of neurodegenerative diseases, were recruited.

### 2.2. Methods

First, pain was measured by a numeric rating scale (NRS), as single-dimensional scale, anchored as 0-no pain and 10-severe pain, to describe overall pain level. NRS score was treated as a continuous variable in the analysis.

Then, pain was evaluated using the Chinese version of the Brief Pain Inventory (BPI), a multidimensional scale [22]. BPI is a structured self-administered qualitative and quantitative questionnaire that provides basic information of pain in the last week, indicating the worst, least, and average perceived pain intensity as well as the pain perceived at the time of the interview (scale from 0, “no pain,” to 10, “pain as bad that you can imagine”). BPI also gives information about the quality of pain, the type and site of pain, and the performed treatments. Patients are also asked to indicate the relief from pain during the last week because of pain treatment on a scale going from 0% (no relief) to 100% (complete relief). Lastly, BPI evaluates the interference of pain with seven daily functions (general activity, mood, walking ability, normal work, relation with other people, sleep, and enjoyment of life) (scale from 0, “does not interfere,” to 10 “completely interferes”). However, because ALS causes the loss of walking ability and interferes with work, these two functions were not considered for the analyses. A Pain Severity Index (PSI) was derived by averaging the following pain severity items: worst and average pain and pain perceived at the time of the interview [3, 23]. Pain degree was defined as no pain (PSI = 0), mild pain (1 ≤ PSI ≤ 3), moderate pain (4 ≤ PSI ≤ 6), and severe pain (7 ≤ PSI ≤ 10). A Pain Interference Index (PII) was derived by averaging the interference of pain on daily functions [3, 23].

Lastly, pain was assessed by neuropathique–4 (DN4) questionnaire (Neuropathic Pain Diagnostic Questionnaire). It includes seven symptoms and three physical examination items. A score of 1 is given to each positive item and a score of 0 to each negative item. Respondents with a total score⩾4/10 are considered to have neuropathic pain [24].

Physical functional status of ALS patients was evaluated with ALS Functional Rating Scale-revised (ALSFRS-R), a 12-item scale assessing various physical functions potentially compromised in ALS. Each item is rated from 0 (worse) to 4 (best), corresponding to a total score ranging from 0 to 48, with higher scores indicating greater physical status and function [25]. Data including gender, age, age at onset, site of onset, and disease duration were collected from all ALS patients.

### 2.3. Statistical Analysis

Comparisons between normal distribution variables or nonnormal distribution variables were performed with Student's *t*-test or Mann–Whitney Test. Frequencies were compared with chi-square. Continuous data are presented as mean ± standard deviation or median/range, and categorical variables are presented as counts or percentages. Correlations were calculated using Pearson's or Spearman's coefficients. All tests were two-tailed. *p* value < 0.05 was considered significant. Analyses were performed with SPSS 25.0.

The study has been approved by the Ethical Committee of our institution, and written informed consent was obtained from each participant.

## 3. Results

### 3.1. Demographic Data

The ALS cohort included 65 patients, 39 males and 26 females, with a mean age of 52.6 years and a mean disease duration of 10 months at the time of the interview. The healthy control cohort included 100 subjects, 61 male and 39 females, with a mean age of 52.7 years. A comparison of demographic data between ALS patients and healthy controls is shown in Table 1.

### 3.2. Prevalence, Site, Description, Severity of Pain, and Treatment

Pain in the preceding week was more frequently reported by patients with ALS (30, 46.2%) than controls (36, 36%) (*p* = 0.193). Twenty-four (80%) of ALS patients with pain considered that the occurrence of pain was related to ALS. Both ALS patients and controls reported pain most frequently at the upper/lower limbs (*p* = 0.015), followed by the trunk. The most used adjectives to describe pain were “sore” (63.3%) and “distending” (63.3%) in ALS patients, while “stinging” (41.7%) and “sore” (36.1%) in controls, all with statistically significance between both groups (*p* < 0.05). In addition, seven ALS patients (23.3%) indicated “tearing” as descriptors, which was absent in controls.

Mean pain score of NRS was 3.1 (SD 1.9) in ALS patients and 4.6 (SD 2.2) in controls (*p* = 0.005). Mean PSI ratings were detected 3.0 (SD 1.7) in ALS patients and 2.9 (SD 1.3) in controls (*p* = 0.688). Seven patients (23.3%) and nine controls (25%) reported moderate pain (4 ≤ PSI ratings ≤ 6) (*p* = 0.875), while none subject was considered severe pain (PSI ≥ 7). Mean pain score of DN4 was 1.2 (SD 1.4) in ALS patients and 1.1 (SD 1.0) in controls (*p* = 0.712); three patients and one control were found to have a DN4 ≥ 4 score (*p* = 0.480). None of them had other pathological conditions (mostly diabetes mellitus, malignancy, paraproteinemia, or vasculitis) known as potential cause of neuropathic pain. They described neuropathic pain as spontaneous numbness, burning, painful cold, or pins-and-needle.

Ten ALS patients (33.3% of those with pain) and twenty-eight controls (77.8% of those with pain) (*p* < 0.001) received a therapy for pain. Five ALS patients (16.7% of those with pain) used pharmacological treatments to relieve pain; six patients (20%) adopted physical therapy for pain relief and only in one patient, with both therapies simultaneously. Nineteen controls (52.8%) used drug therapy or rehabilitation therapy for pain control, respectively; ten subjects adopted both treatment methods. Among ALS patients and controls, nonsteroidal anti-inflammatory drugs (NSAIDs) were the drugs more commonly used for treatment of pain. However, no ALS patients or controls were found to take nonopioid analgesics, opioids, or antiepileptic drugs. Physical therapy, acupuncture, and massage were the most common rehabilitation methods chosen by ALS patients and controls. Details about characteristics and therapy of pain in patients with ALS and controls are given in Table 2.

### 3.3. Clinical Characteristics of ALS Patients with Pain or without Pain

No differences were found in the genders, age at the time of the interview, and site of disease onset between ALS patients with nociceptive pain or without pain (*p* = 0.455, 0.539, and 0.534, respectively). Moreover, ALS patients with nociceptive pain or without pain had a similar disease duration and ALSFRS-R score at the time of the interview (*p* = 0.663 and 0.869, respectively).

Age, ALSFRS-R score in ALS patients with DN4 score⩾4/10 was not different from ALS cases only reporting nociceptive pain; however, ALS patients with a DN4 score⩾4 seemed to have a longer disease duration and a higher PSI and PII score than ALS cases only reporting nociceptive pain (*p* = 0.041, 0.048, and 0.027, respectively) (data not shown).

### 3.4. Association of Pain with ALS Patients' Clinical Characteristics

There was a negative correlation between PSI score and ALSFRS-R score (*r* = −0.398, *p* = 0.029), while no significant correlation between the duration of the disease and PSI ratings was found (*r* = 0.226, *p* = 0.23) (data not shown).

Details about clinical characteristics of ALS patients with pain or without pain are shown in Table 3.

### 3.5. Pain Interference on Daily Functions

Pain mainly interfered with ALS patients' mood (median score of 1), enjoyment of life (median score of 1.5), and the summary score PII (median score of 1.6); other three domains of daily activities were relatively unaffected. The pain interference scores in those domains were significantly worse in ALS patients than in controls (*p* = 0.002, 0.002, and 0.013, respectively).

The areas of general activity, mood, and the summary score PII were correlated with PSI at *p* < 0.05 level in ALS patients cohort, especially in general activity (*p* ≤ 0.001). Details about interference of pain on daily functions in all subjects with pain are shown in Table 4.

## 4. Discussion

Up to now, an increasing number of studies about pain in ALS patients from different countries worldwide have been conducted, with some conflicting results [2, 3, 5–17, 19].

In our case-control study, pain was slightly, but not significantly, more frequent in patients with ALS than in age- and gender-matched controls. The frequency of pain among ALS patients in previous studies varies greatly, from less than 15% up to 85% [1–11, 13–17, 19], which can be explained by the different study designs and settings, different populations and measure instruments or scales, and the number of ALS patients included (range from 7 to 424) [1]. Consistent with previous results, twenty-eight (93.3%) ALS patients in our cohort reported pain most frequently at the upper/lower limbs [3, 7, 15, 17], with a significant difference compared with controls. In a previous ALS-control study, only 10% of controls reported pain in the extremities; however, the number of controls involved in that study was small (46 controls) [7]. The most common types of pain were “sore” (63.3%) and “distending” (63.3%) in ALS patients, a little lower than that of previous results (85.7%, 40 ALS patients) [13]. However, seven patients (23%) described pain as “tearing,” higher than that of previous results [13]. Both “sore” and “tearing” seemed to represent characteristics of secondary pain (mainly nociceptive), which developed in ALS patients as the disease progressed, whereby atrophy and weakness of muscles and prolonged immobility caused degenerative changes in connective tissue, bones, and joints leading to musculoskeletal pain [1].

Though mean pain score of NRS was a little higher in controls than in ALS patients, significant difference in PSI score between groups was not found, in consistent with the PSI score results in previous studies (patients 5.0 (SD 1.8) vs. controls 4.6 (SD 2.6); *p* = 0.09) (patients 3.0 (range 0.5–6.8) vs. controls 2.0 (range 0.5–5.3); *p* = 0.08) [3, 7]. Regarding NRS score was regarded as the pain perceived at the time of the interview, while PSI score was derived by averaging worst, average pain, and pain perceived at the time of the interview. So, the PSI score seemed to be more reasonable and representative for description of pain than NRS. Although severe pain (PSI ≥ 7) was absent in our ALS patient's cohort, seven ALS patients (23.3%) reported moderate pain (4 ≤ PSI ratings ≤ 6), which was in line with previous reports reporting moderate to severe pain in 14–36% of ALS patients [3, 13]. Lastly, only three ALS patients (5%) and one control (1%) presented with neuropathic pain (DN4 score⩾4/10). Previously, only two studies have reported DN4 score⩾4/10 in eight patients (8.6%) and one patient (1%) with ALS from France and Brazil, respectively [10, 11]. Our results further demonstrated ALS patients can have, though rarely, neuropathic pain characteristics.

In contrast to results of previous studies (47%, 70.3% ALS receiving treatment) [3, 7], our ALS patients with pain were undertreated, also less frequently treated than in controls. So, more attention should be paid to identify pain in patients with ALS and to treat it timely and appropriately. Only nonsteroidal anti-inflammatory drugs (NSAIDs), not nonopioid analgesics, opioids, or antiepileptic drugs, were used for the treatment of pain in ALS patients and controls, further providing evidence for the prevalence of nociceptive pain, not neuropathic pain [1]. Compared with previous reports (17%, 11.8%) about massage, acupuncture, and ultrasound for relieving pain [7, 19], physical therapy, acupuncture, and massage were more frequently used by our patients and controls (20% vs. 52.8%), suggesting those traditional rehabilitation therapies seemed more likely to be used by Chinese patients [1].

In line with previous reports, no differences were found in the genders and age at the time of the interview between ALS patients with or without pain [3, 9–11, 13, 14].

Although there was conflicting evidence whether the intensity of pain correlated with disease duration and functional impairment [3, 9–11, 13, 14], ALS patients with or without pain in our cohorts had a similar disease duration and ALSFRS-R score. A previous study found ALS patients with localized pain seemed to present with spinal symptoms at disease onset more frequently, with the bulbar area spared [17]. In our study, up to 83% of ALS patients with pain had a spinal onset, without statistical difference between ALS patients with or without pain, which had been reported in previous studies [11, 14]. Also, consistent with past reports [3, 7], PSI score was negatively correlated with ALSFRS-R score; however, no significant correlation between the duration of the disease and PSI ratings was found. Notably, in contrast with a previous study [11], ALS patients with a DN4 score⩾4 had a longer disease duration and a higher PSI and PII score than ALS cases only reporting nociceptive pain, which have never been reported so far. Whether this reflected a special feature of pain in ALS patients during disease course deserved further study in larger populations and other different countries in the future.

Pain interfered especially with mood and enjoyment of life in ALS patients, and the general activity, mood, and the summary score PII were correlated with PSI in ALS patient's cohort, significantly in general activity, which have been reported before [3, 7, 19].

The first limitation of our study was the relatively small sample size of ALS patients involved (*n* < 100) and the cross-sectional design (instead of a long-term follow-up); therefore, we could not determine the course of pain over time as the disease progressed. Then, the assessment of pain in ALS was mainly limited to nociceptive and neuropathic pain; other causes of pain, including cramp, spasticity, and noninvasive ventilation, were not included in our study. Lastly, incidence of joint contracture, angle of motion at pain site, and neurophysiological examinations were not evaluated, which may be helpful for exploring the mechanism for pain in ALS patients.

## 5. Conclusions

In our study, we comprehensively evaluated pain characteristics and its treatment, interference with daily activities by NRS, BPI, and DN4 in sporadic ALS patients and healthy controls and the correlation with clinical parameters in ALS from Southwestern China, which was the first study about comprehensive evaluation of pain (especially including neuropathic pain assessment) from three different perspectives among ALS patients in China.

Our findings indicated that pain in our ALS cohorts was insufficiently treated and interfered with patients' mood and enjoyment of life. Every effort should be made to identify pain in patients with ALS and to treat it appropriately. Most notably, we found that ALS patients with a DN4 score⩾4 may have a longer disease duration and a higher PSI and PII score than ALS patients reporting nociceptive pain, which has never been reported, strongly deserving further validation in larger samples of ALS patients and in other different countries.

## Figures and Tables

**Table 1:** Comparison between ALS patients and controls and clinical characteristics of patients with ALS.

	ALS patients (*n* = 65)	Controls (*n* = 100)	*p* value
Gender (female : male)	26 : 39	39 : 61	0.9
Age at interview, years (range)	52.6 (30-76)	52.7 (44-68)	0.5
Site of onset (spinal : bulbar)	52 : 13	—	—
Disease duration, months (range)	10 (2-72)	—	—
ALSFRS-R score (range)	40 (21-47)	—	—

ALSFRS-R: Amyotrophic Lateral Sclerosis Functional Rating Scale-revised.

**Table 2:** Characteristics and therapy of pain in patients with ALS and controls.

	ALS patients (*n* = 65)	Controls (*n* = 100)	*p* value
Reporting pain, *n* (%)	30 (46.2%)	36 (36%)	0.193
Localization of pain^a^			
Head/neck (%)	2 (6.7)	5 (13.9)	0.584
Trunk (%)	14 (46.7)	11 (30.6)	0.179
Upper/lower limbs (%)	28 (93.3)	25 (69.4)	0.015
Description of pain^a^		—	
Sore (%)	19 (63.3)	13 (36.1)	0.028
Distending (%)	19 (63.3)	9 (25)	0.002
Stinging (%)	3 (10)	15 (41.7)	0.004
Pain severity			
NRS (SD)	3.1 (1.9)	4.6 (2.2)	0.005
PSI (SD)	3 (1.7)	2.9 (1.3)	0.688
PSI ≥ 4, *n* (%)	7 (23.3)	9 (25)	0.875
DN4 (SD)	1.2 (1.4)	1.1 (1.0)	0.712
DN4 ≥ 4, *n* (%)	3 (5)	1 (1)	0.480
Treatment for pain			
Receiving treatment, *n* (%)	10 (33.3)	28 (77.8)	0.000
Drug therapy, *n* (%)	5 (16.7)	19 (52.8)	0.002
Rehabilitation therapy, *n* (%)	6 (20)	19 (52.8)	0.006

^a^Total is higher than 100% because more sites or description could be indicated. NRS: numerical pain rating scale; PSI: Pain Severity Index; DN4: Douleur Neuropathique-4; SD: standard deviation.

**Table 3:** Clinical characteristics of ALS patients with pain or without pain.

	ALS patients with pain (*n* = 30)	ALS patients without pain (*n* = 35)	*p* value
Gender (female : male)	13 : 17	12 : 23	0.455
Age at interview, year (SD)	51.7 (10.7)	53.1 (6.4)	0.539
Age at onset, year (SD)	50.9 (11.0)	52.5 (11.1)	0.568
Spinal onset, *n* (%)	25 (83)	27 (77)	0.534
Disease duration (range)	11 (2-26)	9 (2-72)	0.663
ALSFRS-R score (range)	41.5 (21-46)	38.97 (21-47)	0.869

ALSFRS-R: Amyotrophic Lateral Sclerosis Functional Rating Scale-revised; SD: standard deviation.

**Table 4:** Interference of pain on daily functions in all subjects with pain.

Pain interference item (range)	All subjects with pain		*p* value
	ALS patients (*n* = 30)	Controls (*n* = 36)	
General activity	0 (0-8)	0 (0-5)	0.171
Mood	1 (0-8)	0 (0-5)	0.002
Relation with other people	0 (0-8)	0 (0-5)	0.133
Sleep	0 (0-8)	0 (0-6)	0.071
Enjoyment of life	1.5 (0-8)	0 (0-7)	0.002
PII	1.6 (0-8)	0 (0-5)	0.013

PII: Pain Interference Index.

## Data Availability

The original data of the current study are available from the corresponding author on reasonable request.
